# A System of Practical Medicine, Comprised in a Series of Original Dissertations

**Published:** 1841-02-13

**Authors:** 


### BIBLIOGRAPHICAL NOTICE.

*A System of Practical Medicine, comprised in a series of Original Dissertations.* Arranged and edited by ALEXANDER TWEEDIE, M. D., F. R. S., Fellow of the Royal College of Physicians, &c. Philadelphia: Lea & Blanchard, 1841.

IT was a happy idea of Dr. Tweedie, to arrange this excellent series of publications in such a form as to make them easy of reference, and at the same time not scattered as in an ordinary cyclopœdia or dictionary. The third volume of the series is certainly superior to any that have yet appeared, with the exception of a very good memoir on inflammation, by

Dr. Thomson, and another on asphyxia, by Mr. Carpenter: the whole volume is contributed by Dr. Williams and Dr. Joy,—that is, the articles on the lungs and their diseases, by the former—those on the heart, by the latter gentleman.

The work of Dr. Williams on Physical Exploration has rendered him well known in this country, and shows that he possesses that practical knowledge of the subject which is necessary to give much value to so important a work as the present one. Hence the articles in the Library are not of course limited to physical exploration, but contain much matter relative to the general symptoms and treatment. The best articles are those on Bronchitis, Laryngitis, and Pulmonary Phthisis. That on pneumonia is good, but not quite equal to the others. The subject of gangrene of the lungs was omitted, or at least only alluded to in a very cursory way; the deficiency has, however, been supplied in the American edition,—additions have also been made to pneumonia, and notes are appended to different chapters of the work.

The memoirs on diseases of the heart by Dr. Joy are, if there be any difference, rather preferable to those of Dr. Williams,—that is, they are more novel, because the previous publications of Dr. Williams had, to a certain extent, rendered us familiar with his views. Taken together, the articles of Dr. Joy are, perhaps, the best work on diseases of the heart: they are condensed, and at the same time are more complete than most other publications, because the subject is brought down to the present time. A number of additional notes are made to these articles.

The extent of the work is too great for a complete analysis of even the leading chapters. To give an idea of the general plan pursued in the volume, and indeed one followed in most volumes of the Library, we will notice more particularly a single article, that on influenza, by Dr. Thomson, which will serve as a specimen of the volume, perhaps a little superior to most of the memoirs.

After a description of the well-known symptoms of the disease, the author proceeds to a very interesting part of the subject—its history.

These are the earlier recorded and least known epidemics of influenza. Others, severe enough to be remembered, appeared in 1775—in '80 at Canton, '81 in Bengal, Europe in '82, travelling from the east. The influenza of 1803 passed from south to north,—disease prevailed at the same time among domestic animals.

In 1832 the disease appeared at Manilla; it reached Great Britain in the spring of 1831. Was at London in June, where the author of this notice experienced a severe attack of it, and prevailed extensively in the United States in the winter of 1831–'2.

In 1830 influenza followed the cholera, beginning in the East: this epidemic prevailed at Paris in April and May, where we suffered like most others at that time in the city; this differed from the preceding epidemic by the great tendency to inflammation of the tonsils.

In 1836–'7 a fatal influenza prevailed in Great Britain; this did not proceed with much regularity from East to West, but it appeared simultaneously in England and on the Baltic.

"*History.* Hippocrates and other ancient authors give slight notices of catarrh resembling the disease under consideration. In latter times the epidemics of 1311, 1323, 1327, 1387, 1400, 1403, 1410, 1414, 1427, 1438, 1482, and 1505, were probably examples of the same affection.

That of 1323 prevailed throughout the whole of Italy, and, according to Buoni Segni, was attributed to a pestilential wind. In 1387 the disorder prevailed at Montpellier and Romagne, and is said by Valesco to have attacked nine-tenths of the population. In 1403, according to Pasquier, a catarrhal visitation occurred at Paris, so severe as to render it necessary to suspend the assizes. The epidemic of 1410, described by Valesco, was characterised by harassing cough, which was regarded as a punishment for singing a licentious song. The visitations of 1414 and 1448 were peculiarly destructive to the aged. Although the short notices now extant of the above epidemics render it extremely probable that they were examples of the disease in question, yet the first instance which we are fully authorised to refer to it, and the first accurately described by medical authors, is that which occurred in the year 1510, and proved fatal to Ann, wife of Philip of Spain. According to Schenck, it was regarded as a new disease. The epidemic proceeding in a north-westerly direction from Malta to Sicily, Spain, Italy, France, and Britain, raged over all Europe, and scarcely missed an individual, but few died except children. The complaint was attended with violent pain over the eye, and with the usual symptoms of more recent attacks. Delirium and gastrodynia were often present, and in some instances, from the seventh to the eleventh day of the attack, snatching of the tendons and syncope occurred. Diarrhœa, or sweating, were common at the decline. It was a frequent practice to apply five blisters, two to the legs, two to the arms, and one to the back of the head. Bleeding and purging are said to have been injurious. A similar disorder prevailed in the autumn of 1557, after a hot dry summer followed by cold northerly winds. The malady was in some places preceded by ill-smelling fogs, and followed by great inundations. The disease took a westerly course from Asia by Constantinople to Europe, and afterwards visited America. The attendant fever, according to Mercatus, exhibited the character of a double tertain. This epidemic was more destructive than that of 1510: 200 persons fell victims to it at Alkmaer in Holland, and 2000 in the small town of Mantua Carpentaria. In the latter instance the mortality was attributed to the employment of bleeding; but many persons of opulence perished under the suspicion of being poisoned. A similar mortality attended the epidemic catarrh of 1580: 9000 died of the disease at Rome, according to Wierus, in consequence of bleeding. The course of the affection was from east and south to west and north. It raged in France after a cold dry wind, following long-continued warm moist weather. A prodigious number of insects covered the roads in France about the time of its appearance. In many places it was observed that animals accustomed to feed on herbs and leaves, took a dislike to their pastures. Birds of passage migrated before the usual time, and those that sleep in low valleys repaired at night to higher districts. (*Salius Diversus de Febre pestilentiali.*) The disorder raged at Sicily in June, at Rome in July; proceeded by Venice and Constantinople to Hungary and Germany, thence to Norway, Denmark, Sweden, and Russia, where it prevailed in December. Although the plague proved very destructive during this year at Cairo, it is remarkable that no European country was visited by that disorder except France, which had been the first to suffer from the catarrhal epidemic.

Bleeding from the nose frequently occurred during this epidemic, but the most characteristic symptoms were vigilance or somnolency, giddiness, resembling that of intoxication, and swelling of the parotid glands. Riverius and Forestus, contrary to the observation of Wierus, speak of bleeding as an important part of the treatment. Sixty thousand persons are reported to have died at Rome in the years 1590 and 1591 of a similar epidemic, associated with severe cerebral symptoms. Another visitation occurred suddenly in April, 1568, and was chiefly prevalent in England, after great extremes of weather: the following summer was exceedingly hot, and a fatal epidemic fever prevailed at its close. In 1663 it is said that 60,000 persons were attacked with influenza in the Venetian states in one week. The disease was attributed by Paulini to an intense fog which came from the Adriatic. In 1669, a similar affection prevailed in Holland, and proved fatal to Sylvius de la Böe. In 1675 Germany was visited in September, and England in October, by a similar epidemic: the previous summer had been unusually warm, and followed by cold moist weather. It is worthy of notice, that the plague prevailed that year in Malta, although it did not visit that place afterwards till 1813. This epidemic was preceded in France by thick fogs. The disease is said by Peu to have been peculiarly fatal to women in the puerperal state. The influenza which prevailed throughout all Europe in 1729 and 1730, was attributed by Hoffmann to changes of weather from heat to cold, and cold to heat, greater than he had ever experienced; by Lœu it was referred to thick sulphurous fogs. Several earthquakes occurred about the same time, and he considered these, as well as the sulphurous transpiration, to be occasioned by the non-occurrence of an eruption of Vesuvius. This catarrhal fever visited every part of Europe in the course of five months, and attacked 50,000 persons at Milan, 60,000 at Rome, and the same number at Vienna. It was very fatal in Paris and London; in the latter place destroying a thousand a week, in September, a greater number of deaths than had occurred in this city in so short a time since the period of the plague. Switzerland suffered little, Italy and Spain very considerably. The disorder generally proved most severe in low marshy situations. In some places it was complicated with petechiæ. Hysterical subjects, when suffering from the epidemic, complained of a peculiar feeling of cold in the course of the sagittal suture. Sanguineous discharges frequently occurred at the termination of the disease, and bleeding was in many instances employed with advantage.

The influences on which catarrhal epidemics depend, appear to have continued in operation from the year 1732 to 1737, and they were associated with remarkable electrical and telluric phenomena. During the spring and autumn of 1732, the weather was unusually dry; the aurora borealis was often peculiarly vivid: volcanic eruptions occurred in various parts of the world; south winds were attended with a dry, and those from the north with a rainy, state of the atmosphere. The disorder is said by Huxham to have ceased suddenly after the explosion of a meteor in the air, which was accompanied with a fetid fog, and produced, for an hour, an appearance as though the north of the heavens was on fire.

It was observed that the epidemic was most apt to be complicated with pectoral affections about the time of the equinoxes, and that cough was generally relieved by diarrhœa, whether occurring spontaneously or produced by purgative medicine. In the autumn of 1732 the disorder overran Europe and visited America. In Britain its course was southerly; it appeared at Edinburgh in November, and did not reach Cornwall till February in the following year. From New England in America it spread southward to Jamaica, Peru, and Mexico. This epidemic affected the intestinal as well as the respiratory system; sanguineous discharges from the nose, lungs, and bowels were frequent, especially when bleeding was omitted. Swelling of the parotid and salivary glands, and of the testes, was occasionally observed. In Edinburgh the poor and those most exposed to atmospheric vicissitudes suffered most. The inmates of the prison and of Heriot's hospital entirely escaped the malady, and although the interments at the Grayfriar's Burial-ground were twice the usual number in the month of January, 1733, yet the fees from the opulent classes did not exceed the common average. Before the eruption of the disease, cough prevailed extensively among horses. The years 1741 and 1742 were remarkable for atmospheric vicissitudes, frequent appearances of the aurora borealis and of meteors resembling soldiers fighting in the air. Catarrhal fever visited several countries during the years 1741 and and 1742; and in the spring of 1743, after five months of excessively severe weather with easterly winds, prevailed generally in Europe under the name of *La grippe*, a word probably derived from *chrypka*, the Polish word for hoarseness. Destructive disease existed at the same time among horses and deer. The influenza commenced with lassitude and shivering, cold hands and feet, pains of head, limbs, and spine, inflamed eyes, loss of taste and appetite. There was a remission of fever at four or five in the evening, and an exacerbation at night. The decline of the disorder was sometimes accompanied with diarrhœa, at other times with an eruption of pustules on the skin. Sauvages describes a hissing noise occurring in the cough of old people affected with the complaint, many of whom died on the ninth or eleventh day. This epidemic preceded the plague in some parts of Sicily. It was less fatal throughout England than in other countries; nevertheless a thousand died of it during one week in London. Epistaxis was a frequent symptom, and in young plethoric subjects bleeding was found useful, and, according to Sauvages, might be repeated with advantage; but Sennertus attributes the destructiveness of the disease at Rome to the injudicious employment of that measure.

The spring of 1762 was characterized by remarkable alternations of intense heat and cold, and by a rapid succession of wind, frost, snow, and rain; and epidemic catarrh was general in Europe. It had however appeared during the previous year in America. The disorder swept away one-third of the inhabitants of Toulon, extended northwards to Breslau, Vienna, and Hamburgh, and in a month passed from London to Edinburgh. This epidemic was singularly capricious in its course and severity, destroying a hundred daily at Breslau, yet sparing Paris and the greater part of France; it was exceedingly mild in many parts of England, especially London, the suberbs of which escaped, but in Norwich was more fatal than the visitation of 1743. It prevailed among the sailors in the Mediterranean in July, during the prevalence of hot weather with easterly winds. Those who were severely attacked usually had either head symptoms, or harassing cough. These symptoms sometimes alternated with each other, but scarcely ever existed together. At the end of the second week at Edinburgh, some of those affected complained of pains of the thigh, others had maniacal attacks. A large proportion of the inhabitants of Europe suffered from the complaint; but scarcely any died, except the old, the asthmatic, and the consumptive. Relapses were frequent, and those who neglected themselves had a tedious cough with some degree of fever, which occasionally was intermittent, and yielded to bark."

This was the last great epidemic of the disease, and was extremely fatal to the aged; to the young, and apparently healthy, it was also productive of much mischief, and in some cases was evidently the forerunner of pulmonary consumption.

"We may deduce from the preceding account that, since medical records have become available, influenza has prevailed on an average once in ten years, and has proved the most destructive of epidemics. There is also reason to believe, that a modified condition of the atmosphere may remain for years after the prevalence of the disease, and occasion a liability to affections of a similar character, to which the term influenzoid might be applied. For eight years after the prevalence of influenza at Lyons, this was found to be the case, 1300 deaths out of 10,096 occurring during that period, being attributed to catarrhal or mucous fevers. We are inclined to believe, that a similar condition has existed in this country since the epidemic of 1833, affections of the bronchi and fauces having been unusually prevalent, associated with severe muscular pains and unusual depression of strength. If this be the case, the subject is one of great importance, and well deserves special investigation."

The diagnosis between influenza and bronchitis of course depends mainly upon the greater development of local symptoms in the latter disease, and the epidemic character of influenza.

The author is, like all others, unsuccessful in his attempts to explain the nature of the disease; it is evidently a subject beyond our reach.

"The danger, in extreme cases of influenza, appears to arise from an excess of mucus proventing the due arterialization of the blood. The difficulty and rapidity of respiration are, however, out of all proportion to the quantity of secretion, or even to the amount of inflammation, and the dyspnœa is sometimes intermittent; and this circumstance cannot easily be explained except by supposing that the cause of the disease must operate, by producing an impression on the vital energy of the lungs, analogous to that occasioned by cutting the nervus vagus; and we may reasonably conjecture that influenza depends on an influence exerted on the nervous system, especially on that part of it having most relation to the bronchial mucous membrane, tending to elicit any latent predisposition to disease, and modified in its character by varieties of constitution as well as by peculiarities of climate and other external conditions."

The sources of the disease are also obscure; it travels, like many other epidemics, especially cholera, from east to west, and is somewhat irregular in its time and mode of outbreak, like other epidemics, but further than this we know little. The explanation of the causes of the disease by referring them to meteorological peculiarities, or to the existence of some noxious matter in the air, is also of little moment.

The treatment is of course very various, for no two epidemics present precisely the same characters. Besides the adoption of the usual mild and almost hygienic remedies, the author recommends a mercurial purge; we confess we do not discover the peculiar virtue of this mineral under these circumstances. Mild diaphoretics are almost always of service; but bleeding, even when the disease is complicated with pneumonia, is generally a doubtful remedy; in fact, the disorder is often of the low or typhoid form, in which bleeding is of little service, or even positively injurious. An inflammation, therefore, complicating the disease, must be treated less actively than if met with under ordinary circumstances. The tincture of lobelia is another remedy recommended.

In our own practice we have relied in most cases nearly on diaphoretics and expectorants, especially on those remedies which have a double action. Thus the infusion of senega and eupatorium combined, and given warm in quantities just enough to produce a very slight nausea, are most effectual; we add to these, local counter-irritants, and frequent mustard foot-baths. We agree with the general opinion that bleeding, though not forbidden, should be prescribed with great caution. If the attack be at all severe, the patient should confine himself to bed.

